# Supplementary material for: Meta-Analysis and Gene Set Enrichment Relative to ER Status Reveal Elevated Activity of MYC and E2F in the “Basal” Breast Cancer Subgroup
Source: PLoS One. 2009 Mar 9;4(3):e4710. doi: 10.1371/journal.pone.0004710 (PMC2650420; doi:10.1371/journal.pone.0004710)
Supplement: Dataset S1 — GSEA results from first screen. The file contains a zipped archive of the HTML results of the second GSEA screen. Reports for enriched or depleted datasets may be accessed from the “index.html” link within the my_analysis.GseaPreranked.1219975950625.rpt.zip ZIP archive. Enrichment plots are available for each of the 50 most enriched and depleted gene sets. (13.32 MB ZIP) [file pone.0004710.s001.zip › my_analysis.GseaPreranked.1219975950625.rpt.zip!//RUIZ_TENASCIN_TARGETS.html]

Details for gene set RUIZ\_TENASCIN\_TARGETS[GSEA]

|  || Dataset | zSco.ERneg.to.ERpos\_collapsed |
| Phenotype | NoPhenotypeAvailable |
| Upregulated in class | na\_pos |
| GeneSet | RUIZ\_TENASCIN\_TARGETS |
| Enrichment Score (ES) | 0.5418488 |
| Normalized Enrichment Score (NES) | 2.1548681 |
| Nominal p-value | 0.0 |
| FDR q-value | 2.6417789E-5 |
| FWER p-Value | 0.0010 |
Table: GSEA Results Summary

  

Fig 1: Enrichment plot: RUIZ\_TENASCIN\_TARGETS      
 Profile of the Running ES Score & Positions of GeneSet Members on the Rank Ordered List

  

| PROBE | GENE SYMBOL | GENE\_TITLE | RANK IN GENE LIST | RANK METRIC SCORE | RUNNING ES | CORE ENRICHMENT || 1 | MCM5 |  |  | 31 | 6.533 | 0.0306 | Yes |
| 2 | CCNA2 |  |  | 52 | 6.297 | 0.0607 | Yes |
| 3 | WNT6 |  |  | 95 | 5.920 | 0.0874 | Yes |
| 4 | CTPS |  |  | 123 | 5.706 | 0.1141 | Yes |
| 5 | MCM4 |  |  | 180 | 5.436 | 0.1372 | Yes |
| 6 | MCM6 |  |  | 198 | 5.317 | 0.1627 | Yes |
| 7 | MCM10 |  |  | 201 | 5.315 | 0.1893 | Yes |
| 8 | ORC1L |  |  | 212 | 5.280 | 0.2151 | Yes |
| 9 | MCM7 |  |  | 289 | 4.970 | 0.2344 | Yes |
| 10 | SCP2 |  |  | 295 | 4.955 | 0.2590 | Yes |
| 11 | EGFR |  |  | 344 | 4.822 | 0.2796 | Yes |
| 12 | CHAF1B |  |  | 479 | 4.411 | 0.2917 | Yes |
| 13 | DMD |  |  | 481 | 4.406 | 0.3138 | Yes |
| 14 | PDGFRA |  |  | 556 | 4.224 | 0.3295 | Yes |
| 15 | RRAS2 |  |  | 628 | 4.081 | 0.3447 | Yes |
| 16 | MCM2 |  |  | 630 | 4.080 | 0.3652 | Yes |
| 17 | MCM3 |  |  | 656 | 4.019 | 0.3835 | Yes |
| 18 | WNT5B |  |  | 664 | 4.011 | 0.4032 | Yes |
| 19 | WNT11 |  |  | 710 | 3.913 | 0.4195 | Yes |
| 20 | FZD9 |  |  | 719 | 3.900 | 0.4385 | Yes |
| 21 | FZD7 |  |  | 767 | 3.811 | 0.4542 | Yes |
| 22 | TYMS |  |  | 829 | 3.738 | 0.4684 | Yes |
| 23 | H2AFX |  |  | 930 | 3.591 | 0.4789 | Yes |
| 24 | CHAF1A |  |  | 971 | 3.525 | 0.4936 | Yes |
| 25 | FZD6 |  |  | 1053 | 3.406 | 0.5047 | Yes |
| 26 | ZYX |  |  | 1345 | 3.088 | 0.4982 | Yes |
| 27 | CDC42EP3 |  |  | 1405 | 3.037 | 0.5091 | Yes |
| 28 | E2F1 |  |  | 1695 | 2.764 | 0.5012 | Yes |
| 29 | SOX4 |  |  | 1758 | 2.720 | 0.5102 | Yes |
| 30 | IFIH1 |  |  | 1819 | 2.680 | 0.5191 | Yes |
| 31 | VCAM1 |  |  | 1849 | 2.649 | 0.5303 | Yes |
| 32 | RRM2 |  |  | 2047 | 2.485 | 0.5279 | Yes |
| 33 | PTPRF |  |  | 2085 | 2.460 | 0.5375 | Yes |
| 34 | MET |  |  | 2188 | 2.394 | 0.5418 | Yes |
| 35 | DHFR /// LOC643509 / |  |  | 2981 | 1.938 | 0.4918 | No |
| 36 | NRG1 |  |  | 2986 | 1.935 | 0.5012 | No |
| 37 | BARD1 |  |  | 3028 | 1.920 | 0.5078 | No |
| 38 | FZD5 |  |  | 3099 | 1.887 | 0.5120 | No |
| 39 | FOSL1 |  |  | 3184 | 1.847 | 0.5150 | No |
| 40 | WNT7A |  |  | 3285 | 1.799 | 0.5165 | No |
| 41 | CCNE2 |  |  | 3424 | 1.738 | 0.5148 | No |
| 42 | WNT1 |  |  | 3495 | 1.712 | 0.5181 | No |
| 43 | HEY1 |  |  | 3698 | 1.621 | 0.5111 | No |
| 44 | FZD3 |  |  | 3728 | 1.612 | 0.5170 | No |
| 45 | WNT10B |  |  | 4085 | 1.478 | 0.4975 | No |
| 46 | TPM1 |  |  | 4482 | 1.324 | 0.4743 | No |
| 47 | SNCAIP |  |  | 4989 | 1.160 | 0.4419 | No |
| 48 | WNT2 |  |  | 5033 | 1.142 | 0.4444 | No |
| 49 | JARID1B |  |  | 5264 | 1.060 | 0.4324 | No |
| 50 | KLF9 |  |  | 5597 | 0.945 | 0.4121 | No |
| 51 | FOXF2 |  |  | 5714 | 0.907 | 0.4079 | No |
| 52 | WNT8B |  |  | 6016 | 0.805 | 0.3892 | No |
| 53 | WNT3 |  |  | 6241 | 0.732 | 0.3760 | No |
| 54 | FZD10 |  |  | 6497 | 0.650 | 0.3600 | No |
| 55 | FZD1 |  |  | 6859 | 0.531 | 0.3354 | No |
| 56 | PLAU |  |  | 7208 | 0.416 | 0.3112 | No |
| 57 | CASK |  |  | 7240 | 0.405 | 0.3109 | No |
| 58 | DKK1 |  |  | 7386 | 0.349 | 0.3017 | No |
| 59 | IL1R1 |  |  | 7459 | 0.325 | 0.2979 | No |
| 60 | WNT2B |  |  | 8585 | -0.119 | 0.2136 | No |
| 61 | FST |  |  | 8866 | -0.240 | 0.1936 | No |
| 62 | ID2 |  |  | 9049 | -0.335 | 0.1816 | No |
| 63 | LEF1 |  |  | 9219 | -0.414 | 0.1709 | No |
| 64 | WNT5A |  |  | 9278 | -0.447 | 0.1688 | No |
| 65 | THBS1 |  |  | 9418 | -0.522 | 0.1609 | No |
| 66 | WNT7B /// LOC647836 |  |  | 10076 | -0.892 | 0.1158 | No |
| 67 | CYR61 |  |  | 10171 | -0.940 | 0.1134 | No |
| 68 | EFEMP1 |  |  | 10437 | -1.112 | 0.0990 | No |
| 69 | TCF12 |  |  | 10495 | -1.160 | 0.1005 | No |
| 70 | NR2F1 |  |  | 10730 | -1.335 | 0.0896 | No |
| 71 | FZD4 |  |  | 11148 | -1.702 | 0.0667 | No |
| 72 | FZD2 |  |  | 11165 | -1.716 | 0.0741 | No |
| 73 | WNT4 |  |  | 11278 | -1.809 | 0.0747 | No |
| 74 | PPAP2A |  |  | 11478 | -2.009 | 0.0698 | No |
| 75 | STC2 |  |  | 11658 | -2.176 | 0.0673 | No |
| 76 | BAZ2B |  |  | 12096 | -2.702 | 0.0479 | No |
| 77 | HBP1 |  |  | 12184 | -2.822 | 0.0555 | No |
| 78 | EDNRA |  |  | 12239 | -2.899 | 0.0660 | No |
| 79 | FOS |  |  | 12389 | -3.085 | 0.0703 | No |
Table: GSEA details [plain text format]

  

Fig 2: RUIZ\_TENASCIN\_TARGETS: Random ES distribution      
 Gene set null distribution of ES for **RUIZ\_TENASCIN\_TARGETS**
